# LiCoPO_4_ cathode from a CoHPO_4_·xH_2_O nanoplate precursor for high voltage Li-ion batteries

**DOI:** 10.1016/j.heliyon.2016.e00081

**Published:** 2016-02-26

**Authors:** Daiwon Choi, Xiaolin Li, Wesley A. Henderson, Qian Huang, Satish K. Nune, John P. Lemmon, Vincent L. Sprenkle

**Affiliations:** Energy and Environment Directorate, Pacific Northwest National Laboratory, Richland, WA99352, USA

**Keywords:** Materials science, Nanomaterials, Materials synthesis, Materials chemistry, Alternative energy technologies

## Abstract

A highly crystalline LiCoPO_4_/C cathode material has been synthesized without noticeable impurities via a single step solid-state reaction using CoHPO_4_·xH_2_O nanoplate as a precursor obtained by a simple precipitation route. The LiCoPO_4_/C cathode delivered a specific capacity of 125 mAhg^−1^ at a charge/discharge rate of C/10. The nanoplate precursor and final LiCoPO_4_/C cathode have been characterized using X-ray diffraction, thermogravimetric analysis − differential scanning calorimetry (TGA-DSC), transmission electron microscopy (TEM), and scanning electron microscopy (SEM) and the electrochemical cycling stability has been investigated using different electrolytes, additives and separators.

## 1 Introduction

Li-ion batteries − widely applied as an energy storage system of choice for electric vehicles, as well as for large scale stationary applications − have the highest energy density amongst the many types of proposed and commercialized rechargeable batteries [1, 2, 3, 4, 5, 6]. Such a high energy density is attained, in part, by both the high specific capacity and voltage of the cathode electrode. Other than the conventionally used oxide-based cathodes, phosphate polyanion-type cathodes have been widely investigated. A notable example is the commercialization of LiFePO_4_ which is one of the most stable cathode materials available due to its unique olivine structure [7, 8, 9, 10, 11, 12]. Among the olivine phosphate-based cathodes (LiMPO_4_: M: Fe, Mn, Co and Ni), LiCoPO_4_ possesses a high redox potential of 4.8 V *vs*. Li/Li^+^, a flat voltage profile, and a high theoretical capacity of 167 mAhg^−1^[13]. However, efforts to utilize LiCoPO_4_ thus far have shown limited capacity and fast fading of the capacity upon repetitive cycles [13, 14, 15]. Like other phosphates, to access the full specific capacity from a LiCoPO_4_ cathode, a nanostructured synthesis of the active material is desired [13, 16, 17, 18].

Various methods have been developed for LiCoPO_4_ cathode synthesis including precipitation, hydrothermal, microwave, solid-state, mechanochemical, supercritical fluid and spray drying [14, 16, 17, 18, 19, 20, 21, 22, 23]. However, many of the synthesis routes reported are not suitable for scale-up and require complicated heat-treatment steps to ensure the formation of pure stoichiometric LiCoPO_4_ since many of the available Co precursor can be easily reduced to form impurities such as Co metal, Co_3_O_4_ and Li_3_PO_4_ phases. Previously, NH_4_CoPO_4_ nanoplates were used as a starting material for LiCoPO_4_, but multiple heat-treatments in both air and inert atmosphere were required to ensure formation of stoichiometric LiCoPO_4_ since H_2_ produced during the decomposition of NH_4_CoPO_4_ generates Co metal [14]. Other metal organic compounds are also prone to produce Co metal during heat-treatment by carbothermal reduction.

To form a stoichiometric LiCoPO_4_ cathode without impurities, precursor compounds with strong Co-P bonding are desired. In the present work, a nanostructured CoHPO_4_·xH_2_O precursor was used to simplify the synthesis process and to minimize impurities. Previously, in related work, a CoHPO_4_·3H_2_O nanosheet electrode was hydrothermally synthesized for supercapacitors applications [24]. Finally, the effect of the electrolyte and separator on the cycling stability of the LiCoPO_4_/C cathode obtained was investigated.

## 2 Experimental

The LiCoPO_4_ cathode was synthesized by a solid-state reaction using LiOH, Ketjen black carbon (AkoNobel) and CoHPO_4_·xH_2_O nanoplate precursors. The CoHPO_4_·xH_2_O nanoplates were synthesized using a simple precipitation route from disodium pyrophosphate (NaH_2_P_2_O_7_: Aldrich) and cobalt acetate tetrahydrate (Co(CH_3_COO)_2_·4H_2_O: Aldrich) in DI-water. Initially, 9.12 g of ammonium acetate (NH_4_C_2_H_3_O_2_: Aldrich) and 8.18 g of Na_2_H_2_P_2_O_7_ were dissolved in 200 ml of DI-water, while 18.43 g of Co(CH_3_COO)_2_·4H_2_O was dissolved separately in 100 ml of DI-water. The cobalt solution was slowly added to the disodium pyrophosphate solution while stirring and the pH of the mixture reached a value of 5 ∼ 6. After the reaction proceeded for 8 h at 80 °C, the precipitated CoHPO_4_·xH_2_O was collected by centrifuging the solution and the solid was washed several times with DI-water and ethanol. The obtained CoHPO_4_·xH_2_O powder was dried at 80 °C in an oven for 2 days. The degree of hydration of the CoHPO_4_·xH_2_O was measured via TGA giving x equal to 1 (i.e., CoHPO_4_·H_2_O) for the precursor dried at 80 °C in air. For the final LiCoPO_4_/C cathode synthesis, the CoHPO_4_·H_2_O and LiOH (molar ratio 1:1) were mixed with 4.04 wt% (i.e., 5 wt% relative to LiCoPO_4_) of Ketjen black using a planetary mill (Retch 200CM) for 4 h followed by heat-treatment in a tube furnace at 700 °C for 10 h under an UHP-Ar atmosphere with a heating rate of 5 °C min^−1^.

A simultaneous differential scanning calorimetry (DSC) and thermogravimetric analysis (TGA) system (Netzsch STA 449C Jupiter) equipped with a SiC high temperature furnace (25–1550 °C) and a type-S sample holder was used to study the dehydration and phase transformation of the CoHPO_4_·xH_2_O nanoplate precursor. The powder sample was heated in an air environment up to 700 °C at a ramp rate of 5 °C min^−1^. The crystal structure of the as-prepared LiCoPO_4_/C composite was determined by X-ray diffraction (XRD) using a Rigaku Mini-Flex II with a CuKα sealed tube (λ = 1.54178 Å). All of the samples were scanned in a 2θ range between 5 to 80°, with a step size of 0.01° and an exposure time of 30 s. A JEOL 7001F scanning electron microscope (SEM) system was used to investigate the particle morphology. A high-resolution transmission electron microscopic (HRTEM) analysis was conducted using a FEI Tecnai G2 microscope with an acceleration voltage of 200 kV.

Electrodes were prepared by casting a slurry of the LiCoPO_4_/C composite, acetylene black (MTI), and polyvinylidenedifluoride (PVDF, MTI) in *N*-methylpyrrolidone (NMP: Aldrich) solvent onto an Al foil current collector. The total weight percentage of carbon and PVDF in the electrode was 10 wt% (final weight ratio of LiCoPO_4_: carbon: PVDF was 8:1:1). After drying at 120 °C overnight under vacuum, the electrodes were punched into 1.6 cm^2^ disks. The active material loading was 1 ∼ 2 mg cm^−2^. Pure Li metal was used as an anode in a 2325 coin cell (NRC). The electrolyte consisted of 1 M LiPF_6_ in a mixture of dimethyl carbonate (DMC) and ethylene carbonate (EC) (1:1 volume ratio) or DMC and fluoroethylene carbonate (FEC) (4:1 volume ratio) with 1.5 wt% of trimethylboroxine (TMB) additive. A Celgard 2500 or glassy microfiber (Whatman) separator was used. The coin cells were assembled in an Ar-filled MBraun glove box. The electrochemical tests were performed on an Arbin BT-2000 battery cycler at room temperature. The cells were cycled between 3.0 and 5.2 V *vs.* Li/Li^+^ at a C/10 (1C = 167 mAhg^−1^) rate unless otherwise noted in the rate capability comparison.

## 3 Results and discussion

**Fig. 1:**
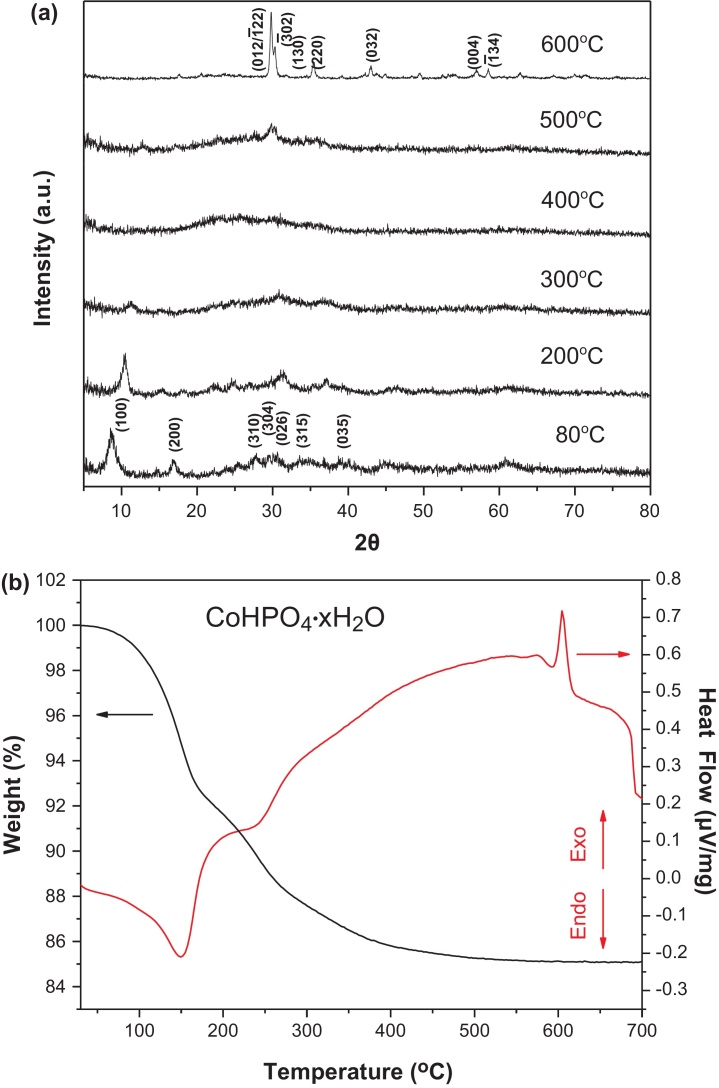
(a) XRD patterns of the CoHPO_4_·xH_2_O nanoplate precursor at various temperatures in an air atmosphere and (b) TGA-DSC analysis of the CoHPO_4_·xH_2_O nanoplate precursor in an air atmosphere with a heating rate of 5 °C min^-1^.

To synthesize the LiCoPO_4_ nanoparticles, the CoHPO_4_·xH_2_O nanoplate precursor was obtained by a precipitation reaction between Co^2+^ and P_2_O_7_^4−^ (from Na_2_H_2_P_2_O_7_) in acidic media of pH 5 ∼ 6 at 80 °C for 8 h resulting in a violet CoHPO_4_·xH_2_O powder. Fig. 1(a, b) shows the powder XRD patterns of the as-prepared CoHPO_4_·xH_2_O precursor at different heat-treatment temperatures and the TGA-DSC analysis to determine the H_2_O content up to 600 °C in air. All of the indexed peaks in the pattern are in agreement with CoHPO_4_·3H_2_O (JCPDS no. 39–0702) at 80 °C. No peaks of other phosphites or phosphates were detected from these patterns. The broad peaks indicate the presence of nanostructured or defected nature of the as-prepared CoHPO_4_·xH_2_O samples making them suitable for the final LiCoPO_4_ nanoparticles with better electrochemical performance. The continuous dehydration of the samples upon increasing temperature resulted in a composition close to CoHPO_4_·1.5H_2_O (JCPDS no. 22–0222) at 200 °C followed by amorphization above 200 °C up to 500 °C in air. At 600 °C, the well-defined diffraction peaks (peak position and their relative intensities) were clearly observed and successfully indexed to the reflections of the monoclinic α-Co_2_P_2_O_7_ crystal structure (JCPDS no. 49–1091) with a space group of P2_1_/c and cell parameters of *a* = 8.924, *b* = 8.366, and *c* = 9.016. Moreover, no other discernable diffraction reflections corresponding to other impurities (e.g., Co_2_O_3_, Co_3_O_4,_ etc.) at 600 °C, indicating the Co/P ratio is 1/1 for the as-prepared CoHPO_4_·xH_2_O precursor.

For the subsequent stoichiometric LiCoPO_4_ synthesis, an accurate determination of the H_2_O content present in the as-prepared CoHPO_4_·xH_2_O precursor needed to be determined to calculate the stoichiometric amount of LiOH required. Therefore, a TGA-DSC analysis was performed on the as-prepared CoHPO_4_·xH_2_O where a 14.93 wt% decrease in weight was observed from 80 °C to 600 °C which is equivalent to x = 1 (15.63 wt% decrease) when a single Co_2_P_2_O_7_ phase at 600 °C was used as a standard. The TGA result indicated a lower H_2_O content (x = 1) than the XRD result where a crystal structure close to x = 3 was observed. The discrepancy between the XRD and TGA results for the hydration level of CoHPO_4_·xH_2_O is likely due to the creation of defects with a lower crystallinity thereby showing broader peaks since it has been reported that the dehydration of CoHPO_4_·xH_2_O (0.5 ≤ x ≤ 1.5) occurs almost isothermally which is sensitive to the synthesis temperature, drying condition, moisture level and synthesis time [25].

From both XRD and TGA studies, the crystalline CoHPO_4_·H_2_O dehydrates to amorphous CoHPO_4_ as the temperature is increased above 200 °C. Between 200 °C and 500 °C, the amorphous CoHPO_4_ slowly dehydrates to amorphous Co_2_P_2_O_7_ before the start of crystallization above 590 °C. At 600 °C, β-Co_2_P_2_O_7_ is the stable phase, but transforms to α-Co_2_P_2_O_7_ as the temperature is decreased to room temperature [25, 26]. Overall, the dehydration and phase evolution of the CoHPO_4_·H_2_O can be described as follows:

$$CoHPO_{4}\cdot H_{2}O\;\;\overset{∼\;200\;{}^{\circ}C}{\underset{-xH_{2}O}{\to }}\;CoHPO_{4}\cdot 0.2H_{2}O\;\overset{>250\;{}^{\circ}C}{\underset{-xH_{2}O}{\to }}\;amorphous\;CoHPO_{4}\;\overset{>\;500\;{}^{\circ}C}{\underset{-H_{2}O}{\to }}\;amorphous\;\;Co_{2}P_{2}O_{7}\;\overset{>\;590\;{}^{\circ}C}{\underset{crystallization}{\to }}\;\beta -Co_{2}P_{2}O_{7}\;\overset{∼\;300\;{}^{\circ}C}{\underset{reversible\;transformation}{\to }}\alpha -Co_{2}P_{2}O_{7}$$

**Fig. 2:**
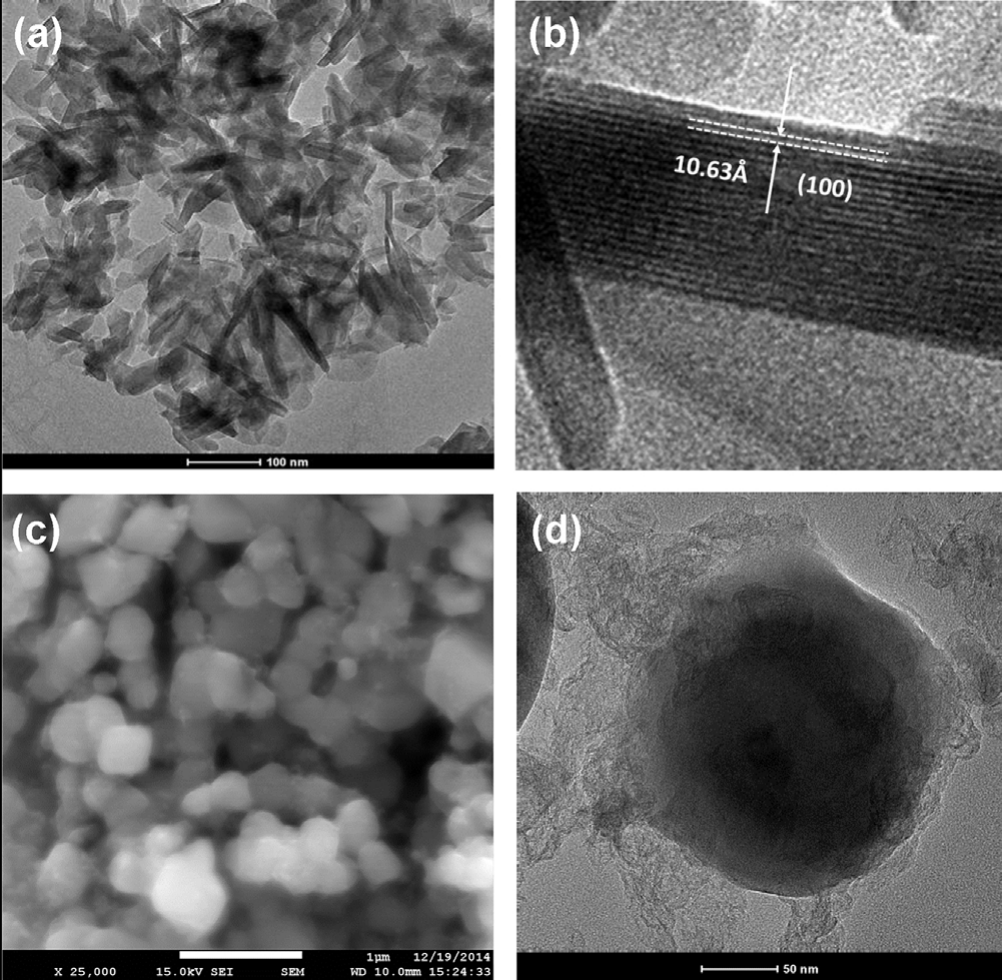
High-resolution (a, b) TEM images of the synthesized CoHPO_4_·H_2_O nanoplate precursor, (c) SEM and (d) TEM images of the LiCoPO_4_/C synthesized at 700 °C under an UHP-Ar atmosphere.

Fig. 2 shows the SEM and TEM images of the as-prepared CoHPO_4_·H_2_O and the final LiCoPO_4_/C obtained at 700 °C. A typical low-magnification TEM image in Fig. 2(a) shows a thin CoHPO_4_·H_2_O nanoplate morphology in the 2D microscale with 10 ∼ 20 nm thickness and ∼100 nm width and length. From the HRTEM image of the edge of a CoHPO_4_·H_2_O nanoplate comprised of ∼20 single layers in Fig. 2(b), the measured distance of the neighboring lattice fringes was 10.63 nm which corresponds to the major (100) plane (interlayer spacing of 10.7 nm) of CoHPO_4_·3H_2_O indicating a layered structure for the CoHPO_4_·H_2_O. The slightly lower spacing is probably due to a lower H_2_O content from defects. In contrast, the synthesized final LiCoPO_4_/C consists of spherical particles 100 ∼ 400 nm in size covered with carbon. The LiCoPO_4_ is obtained at 700 °C via the proposed reaction:

$$LiOH\;+\;CoHPO_{4}\cdot xH_{2}O\;\overset{∼\;700\;{}^{\circ}C}{\to }\;LiCoPO_{4}\;+\;(1\;+x)\;H_{2}\;O↑$$

The layered structure of the CoHPO_4_·xH_2_O nanoplates and the amorphization at elevated temperature facilitate the Li diffusion into the CoHPO_4_·xH_2_O matrix with only H_2_O as a by-product resulting in uniform nanoparticles without much grain growth.

**Fig. 3:**
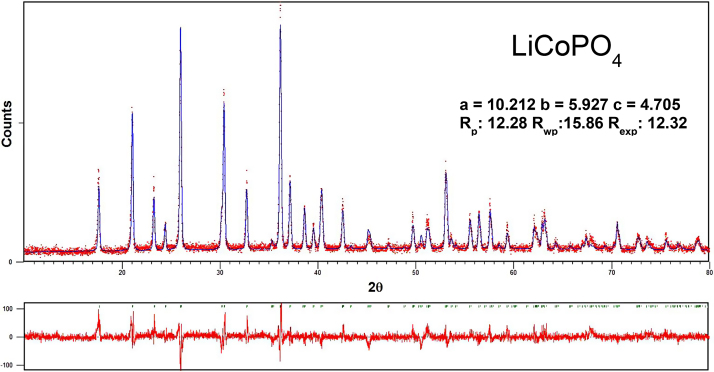
Rietveld refinement of the XRD pattern of the LiCoPO_4_/C cathode.

Fig. 3 shows the Rietveld refinement of the XRD pattern of the LiCoPO_4_/C nanocomposite based on the orthorhombic *Pnma* space group where the *b* and *a* axes were switched from *Pnmb* (JCPDS No. 33–0804), which is isostructural to LiCoPO_4_. The refined lattice parameter matches closely that of pure orthorhombic LiCoPO_4_ (*Pnma*, *a =* 10.212, *b* = 5.927, *c =* 4.705 Å). Moreover, no other discernable diffraction reflections were evident corresponding to other impurities known to be present from heat-treatment with carbon during LiCoPO_4_/C synthesis, indicating the stoichiometric nature of the CoHPO_4_·H_2_O nanoplate precursor.

**Fig. 4:**
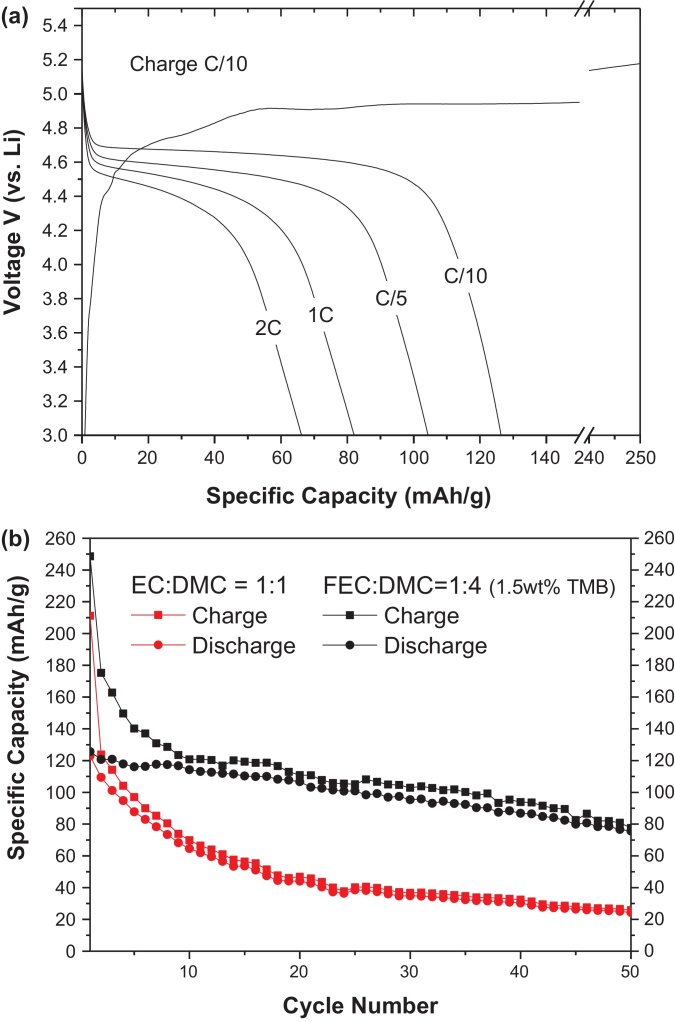
(a) Electrochemical charge–discharge curves at various C-rates and (b) cycling performance of the LiCoPO_4_/C cathode using different electrolytes and separators at a C/10 charge-discharge rate.

Fig. 4(a) shows the voltage profiles of the LiCoPO_4_/C cathode for various discharge rates. At a C/10 rate, a specific capacity of 125 mAhg^−1^ was observed and at a 1C rate, a specific capacity of > 80 mAhg^−1^ was achieved. The rate performance of LiCoPO_4_ is better than that of a LiMnPO_4_ cathode [8].

While the high voltage LiCoPO_4_ cathode delivers an acceptable capacity and rate performance without the need of excessive conductive carbon (as is done for a LiMnPO_4_ cathode), LiCoPO_4_ has been reported to have a fast fade in capacity upon electrochemical cycling which limits its application. Numerous reports on the origin of the poor cycling stability of LiCoPO_4_ indicate that the fast capacity fading in LiPF_6_ containing electrolyte solutions is believed to be due to the nucleophilic attack of the HF (always) present in these electrolyte on the P atoms of the olivine compound in the delithiated state resulting in the formation of soluble PO_3_F^2-^, PO_2_F_2_^−^, POF_3_ and H_2_O. The H_2_O produced then reacts with PF_6_^−^, POF_3_ and PO_2_F_2_^−^ to produce more HF [15, 27]. Therefore, to prevent CoPO_4_ dissolution during cycling, HF should ideally be eliminated − which is a challenging task. Various strategies have been tested to stabilize the cycling performance including the use of an HF scavenging separator, protective coating, and doping to induce SEI (solid electrolyte interphase) layer formation using electrolyte additives [15, 28]. Using the latter approach, Fe-substituted LiCoPO_4_ exhibited an improved cycling stability due to the stabilization of the structure in the delithiated state [29, 30]. However, a lower specific capacity was achieved when Fe was used as a dopant. Recently, various electrolyte additives have also improved the cycling performance. An improved capacity retention was observed when LiCoPO_4_ was cycled with an electrolyte containing either tris(hexafluoroisopropyl) phosphate (HFiP) or trimethylboroxine (TMB) [31]. Additionally, the use of alternative separators such as glassy paper or quartz has increased the cycling stability relative to the conventional polyethylene(PE)/polypropylene (PP) separators due to the presence of silica, which is known to be a HF scavenger.

The cyclic performance of the LiCoPO_4_/C cathode is shown in Fig. 4(b). The large irreversible losses in the capacities observed at the beginning of the cycles are believed to be due to the SEI layer formation by the decomposition of the electrolyte and the additives. When a conventional 1 M LiPF_6_ in EC:DMC (1:1 v/v) electrolyte was used, ∼50% and ∼80% degradation in the specific capacity after 10 and 50 cycles has been observed, respectively, which is similar to previous reports [14, 28]. However, when a glassy separator and a 1 M LiPF_6_ in FEC:DMC (1:4 v/v) electrolyte with 1.5 wt% TMB additive was used, over 90% and 60% of the initial capacity has been retained after 10 and 50 cycles, respectively. The FEC-based electrolyte with the TMB additive demonstrates a dramatic improvement in the cycling characteristics of the LiCoPO_4_/Li cells as compared to the EC-based electrolyte. Achieving an electrolyte with high voltage stability and HF minimization is a challenging task. Further investigations on the cycling stability of the cathode are currently ongoing and detailed information regarding the influence of the electrolyte formulations on the LiCoPO_4_ cycling stability will be reported in the future.

## 4 Conclusions

A highly stoichiometric LiCoPO_4_/C cathode material has been synthesized using a CoHPO_4_·xH_2_O precursor obtained by a simple precipitation route at room temperature which is suitable for a large scale synthesis. The CoHPO_4_·xH_2_O obtained has a nanoplate shape morphology with a x = 1 hydration level. A pure, stoichiometric LiCoPO_4_/C cathode was obtained by a single step heat-treatment at 700 °C which delivers a specific capacity of 125 mAhg^−1^ at a C/10 rate containing 10 wt% conductive carbon additive indicating that the CoHPO_4_·xH_2_O precursor is an ideal starting material for LiCoPO_4_ cathode synthesis. With a variation in the composition of a carbonate-based electrolyte and use of an additive, a significant improvement in the cycling stability was observed. It is likely that, with a more systematic understanding of the degradation mechanism(s) and further electrolyte optimization, the cycling performance of the high voltage LiCoPO_4_ cathode can be significantly improved.

## Declarations

### Author contribution statement

Daiwon Choi: Conceived and designed the experiments; Wrote the paper.

Daiwon Choi, Qian Huang, Wesley A. Henderson, Xiaolin Li: Performed the experiments.

Daiwon Choi, Satish K. Nune: Analyzed and interpreted the data.

John P. Lemmon, Vincent L. Sprenkle: Contributed reagents, materials, analysis tools or data.

### Funding statement

This work was supported by the U.S. Department of Energy's (DOE's) Office of Electricity Delivery and Energy Reliability (OE) (under Contract No. 57558).

### Competing interest statement

The authors declare no conflict of interest.

### Additional information

No additional information is available for this paper.
